# Editorial: Natural Compounds in Food Safety and Preservation

**DOI:** 10.3389/fnut.2021.759594

**Published:** 2021-09-16

**Authors:** Susana Ferreira, Gloria Sanchez, Maria J. Alves, Maria J. Fraqueza

**Affiliations:** ^1^CICS-UBI – Health Sciences Research Centre, University of Beira Interior, Covilhã, Portugal; ^2^Department of Preservation and Food Safety Technologies, Instituto de Agroquímica y Tecnología de Alimentos – Consejo Superior de Investigaciones Científicas (IATA-CSIC), Valencia, Spain; ^3^Centro de Investigação de Montanha (CIMO), Instituto Politécnico de Bragança, Bragança, Portugal; ^4^CIISA-Centro de Investigação Interdisciplinar em Sanidade Animal, Faculdade de Medicina Veterinária, Universidade de Lisboa, Lisboa, Portugal

**Keywords:** editorial, natural compounds, food safety, food preservation, antimicrobial

Food safety is a global challenge, with foodborne diseases posed as a relevant concern for human health, and food microbial spoilage being a problem for agri-food companies (1). Considerable research has been dedicated to diverse approaches that can be applied to control foodborne pathogens and microbial spoilage, among which the potential use of natural compounds has been highlighted as a strategy for improving food safety, but also quality and extending self-life (1–3). Furthermore, the negative consumer perception of the synthetic preservatives used in food industry, associated with an increasing demand for maintenance of nutritional and quality properties, has encouraged the pursue for the use of natural-based preservatives in food production (1–3).

In this context, in this Research Topic, natural antimicrobial compounds have been highlighted by their activity against *Chronobacter* spp. in infant powdered formula by Yemiş and Delaquis. The authors reviewed the potential of natural compounds from plants, microbial and animal sources as alternatives to synthetic chemical preservatives, addressing nutritional, toxicological, and regulatory issues. In fact, the use of natural antimicrobial compounds needs to be guided considering the regulatory framework, and so the authors suggest the use of well-studied single compounds over multiple-component preparations.

Among the natural compounds, essential oils have been pointed as promising antimicrobial mixtures. Yousefi et al. reviewed the potential application of essential oils with antilisterial activity in meat and poultry products, since contaminated meat products are recognized as one main source for *Listeria monocytogenes* infection. The authors described the efficiency of several essential oils in the control of *L. monocytogene*s, whilst addressing the mechanism of action of some selected compounds and the major drawbacks associated with the application of essential oils in food products. The activity of natural compounds in food is dependent of various factors, namely on the complexity and composition of the product, this highlights the need of the validation of antimicrobial activity in food matrixes. Kiprotich et al. explored the use of thyme oil combined with *Yucca schidigera* extract to marinate raw chicken breast meat in lemon juice. The authors considered the potential of antimicrobial marinade formulations as an approach to reduce enteric pathogens. Based on their results, thyme oil showed to be an enhancer of the inactivation of *Salmonella enterica* on raw chicken breast, increasing the antimicrobial efficacy of lemon juice marinade containing yucca extract to emulsify the thyme oil.

Besides, the perspective of the use of natural antimicrobial compounds for controlling foodborne pathogens, research has also been carried out to elucidate its use against microbial spoilage. Shen et al. reported the antifungal activity of Loquat leaves extract against citrus postharvest pathogens, providing a mechanistic overview of the anti-*Penicillium digitatum* activity. The antifungal activity of this extract against *P. digitatum* was attributed to the derangement of cell membrane permeability and the disordered energy metabolism.

Among the limitations of the use of essential oils are: off-flavors and odors that may result in an unacceptance of the food product by the consumer usually associated with the use of high concentrations of essential oils, the degradation of the components or the limited interaction with the microorganisms. The incorporation of these natural bioactive compounds, into edible coatings, food packaging materials, or other formulations may be presented as an approach to overcome these problems. Asensio et al. reported the use of nanoemulsions as an approach to encapsulate, protect, and deliver Argentinean oregano essential oil. The authors optimized the physical stability of the nanoemulsion and characterized it, showing that the formulation may even increase the antimicrobial activity and inhibition of quorum sensing when comparing with the pure essential oil.

The application of these natural compounds may be accomplished alone or in combination with already existing preservatives or even processing methods for the development of a food preservation system, providing mechanisms to ensure food safety. This subject was approached by Barroug et al. who reviewed the use of natural compounds with non-thermal strategies on poultry products addressing the effects on the microbiological and physicochemical characteristics. This paper gives a well-balanced overview of the use of non-thermal technologies, natural compounds and their combination as an intervention for safer poultry products.

In conclusion, the present Research Topic provides several examples of natural antimicrobial compounds and their application in different contexts.

## Author Contributions

SF drafted the manuscript. GS, MA, and MF provided critical review and insight and revised the final version of the editorial. All authors contributed to the article and approved the submitted version.

## Conflict of Interest

The authors declare that the research was conducted in the absence of any commercial or financial relationships that could be construed as a potential conflict of interest.

## Publisher's Note

All claims expressed in this article are solely those of the authors and do not necessarily represent those of their affiliated organizations, or those of the publisher, the editors and the reviewers. Any product that may be evaluated in this article, or claim that may be made by its manufacturer, is not guaranteed or endorsed by the publisher.
